# Mcl-1 inhibition overcomes intrinsic and acquired regorafenib resistance in colorectal cancer

**DOI:** 10.7150/thno.45363

**Published:** 2020-07-09

**Authors:** Xiangping Song, Lin Shen, Jingshan Tong, Chaoyuan Kuang, Shan Zeng, Robert E. Schoen, Jian Yu, Haiping Pei, Lin Zhang

**Affiliations:** 1 UPMC Hillman Cancer Center, Pittsburgh, PA 15213, USA.; 2 Department of Gastrointestinal Surgery, Xiangya Hospital, Central South University, Changsha, Hunan, 410008, P.R. China.; 3 Department of Pharmacology and Chemical Biology, University of Pittsburgh School of Medicine, Pittsburgh, PA 15213, USA.; 4 Department of Oncology, Xiangya Hospital, Central South University, Changsha, Hunan, 410008, P.R. China.; 5 Department of Medicine, University of Pittsburgh School of Medicine, Pittsburgh, PA 15213. USA.; 6 Department of Pathology, University of Pittsburgh School of Medicine, Pittsburgh, PA 15213. USA.

**Keywords:** Mcl-1, FBW7, regorafenib, colorectal cancer, apoptosis

## Abstract

Intrinsic and acquired resistance to targeted therapies is a significant clinical problem in cancer. We previously showed that resistance to regorafenib, a multi-kinase inhibitor for treating colorectal cancer (CRC) patients, can be caused by mutations in the tumor suppressor *FBW7*, which block degradation of the pro-survival Bcl-2 family protein Mcl-1. We tested if Mcl-1 inhibition can be used to develop a precision combination therapy for overcoming regorafenib resistance.

Methods: Small-molecule Mcl-1 inhibitors were tested on CRC cells with knock-in (KI) of a non-degradable Mcl-1. Effects of Mcl-1 inhibitors on regorafenib sensitivity were determined in *FBW7*-mutant and -wild-type (WT) CRC cells and tumors, and in those with acquired regorafenib resistance due to enriched *FBW7* mutations. Furthermore, translational potential was explored by establishing and analyzing *FBW7*-mutant and -WT patient-derived organoid (PDO) and xenograft (PDX) tumor models.

Results: We found that highly potent and specific Mcl-1 inhibitors such as S63845 overcame regorafenib resistance by restoring apoptosis in multiple regorafenib-resistant CRC models. Mcl-1 inhibition re-sensitized CRC tumors with intrinsic and acquired regorafenib resistance *in vitro* and *in vivo,* including those with *FBW7* mutations. Importantly, Mcl-1 inhibition also sensitized *FBW7*-mutant PDO and PDX models to regorafenib. In contrast, Mcl-1 inhibition had no effect in *FBW7*-WT CRCs.

Conclusions: Our results demonstrate that Mcl-1 inhibitors can overcome intrinsic and acquired regorafenib resistance in CRCs by restoring apoptotic response. *FBW7* mutations might be a potential biomarker predicting for response to the regorafenib/Mcl-1 inhibitor combination.

## Introduction

Colorectal cancer (CRC) is the second leading cause of cancer-related deaths in the US 1. Metastatic, chemotherapy-refractory CRC patients are treated with targeted drugs such as the approved multi-kinase inhibitor regorafenib 2. However, most CRCs are not responsive to regorafenib treatment 2, and CRCs treated with targeted therapies almost invariably develop resistance shortly after initial therapy 3, 4. Understanding the mechanisms of intrinsic and acquired resistance and developing rational combination therapies for overcoming such resistance is pivotal for improving efficacy of targeted therapies.

Regorafenib inhibits the RAS/RAF/MEK/ERK axis, which is aberrantly activated in most CRCs due to prevalent *RAS* and *RAF* mutations 5. We recently showed that the therapeutic activity of regorafenib in CRC cells is associated with apoptosis induction and proteasomal degradation of myeloid cell leukemia 1 (Mcl-1), a pro-survival Bcl-2 family protein 6. CRC cells that contain mutations in *F-box and WD repeat domain-containing 7* (*FBW7*) are intrinsically resistant to regorafenib. FBW7 is a tumor suppressor and E3 ubiquitin ligase frequently mutated in CRCs 7, 8. In response to regorafenib treatment, FBW7 binds to phosphorylated Mcl-1 and promotes Mcl-1 ubiquitination and subsequent degradation 9. Blocking Mcl-1 phosphorylation by a knock-in approach abrogates Mcl-1 binding to FBW7. This subsequently leads to Mcl-1 stabilization, which suppresses regorafenib-induced killing of CRC cells 9. Importantly, CRC cells with acquired regorafenib resistance were found to have blocked Mcl-1 degradation and enriched *FBW7* hot-spot mutations at R505, R465 and R479 6. The critical role of Mcl-1 stabilization and *FBW7* mutations in intrinsic and acquired resistance to regorafenib suggests that Mcl-1 is an attractive target for developing a precision combination therapy for overcoming regorafenib resistance in *FBW7*-mutant CRCs.

Targeting pro-survival Bcl-2 family proteins is a promising therapeutic strategy and has led to the approval of the Bcl-2-selective inhibitor Venetoclax 10. A number of small-molecule Mcl-1 inhibitors with distinct chemical structures have been described, among which the most promising ones include S63845, AMG176, AZD5991, and VU661013 11-14. However, most of the preclinical and clinical studies on the Mcl-1 inhibitors have been carried out on hematopoietic malignancies in which Mcl-1 by itself is required for maintenance of cell survival 11-14. The rationale and efficacy for targeting Mcl-1 in solid tumors such as CRC have yet to be demonstrated, in part due to lack of biomarkers for predicting response to Mcl-1 inhibition.

In this study, we evaluated the effects of Mcl-1 inhibitors on regorafenib sensitivity by using isogenic cell line, xenograft, patient-derived organoid (PDO) and patient-derived xenograft (PDX) models. Our results revealed a striking efficacy of Mcl-1 inhibitors in sensitizing regorafenib-resistant CRCs to regorafenib. We demonstrate the critical role of *FBW7* mutations in promoting Mcl-1-dependent resistance to regorafenib, as well as how this resistance can be reversed by Mcl-1 inhibition. Our study provides a compelling rationale for developing a precision combination therapy on CRCs that are refractory to regorafenib treatment using Mcl-1 inhibitors.

## Materials and Methods

### Cell culture

Human CRC cell lines, including HCT116, DLD1, Lim1215, Lim2405, RKO, SW837, SW48, LoVo, SW1463, HCT-8 and LS411N, and the mouse CRC cell line CT26 were obtained from ATCC. HCT116 cells with knock-in of the Mcl-1 phosphorylation site mutant S121A/E125A/S159A/T163A (*Mcl-1*-KI) were generated by homologous recombination as described previously 9. Regorafenib-resistant HCT116 (HCT116-R) and Lim1215 (Lim1215-R) were generated by 4 cycles of regorafenib selection as previously described 6. Isogenic *FBW7*-KO HCT116 cells was obtained from Horizon Discovery 7. All cell lines were authenticated by genotyping and analysis of protein expression by western blotting throughout the study, and also checked for mycoplasma contamination by PCR. Cell lines were maintained at 37°C and 5% CO_2_ in McCoy's 5A modified media (Invitrogen) supplemented with 10% defined FBS (HyClone), 100 U/mL penicillin, and 100 mg/mL streptomycin (Invitrogen). For drug treatment, cells were plated in 12-well plates at 20-30% density 24 hours before treatment. Anticancer agents including regorafenib, sorafenib, S63845, AZD5991, and AMG176 were dissolved in dimethylsulfoxide (DMSO) and diluted to appropriate concentrations with cell culture medium.

### Analysis of cell viability

Cells seeded in 96-well plates at a density of 1×10^4^ cells/well were treated with regorafenib at different concentrations +/- Mcl-1 inhibitor for 72 hours. Cell viability was analyzed by MTS (3-(4, 5-dimethylthiazol-2-yl)-5-(3-carboxymethoxy-phenyl)-2-(4-sulfophenyl)-2H-tetrazolium) assay using the MTS Assay Kit (Promega) as described 6. Cells plated in 12-well plates at 20-30% confluency were treated with regorafenib +/- Mcl-1 inhibitor at different concentrations for 48 hours, followed by staining of viable cells by crystal violet as described 15. MTS and crystal violet staining results were quantified using a WallacVictor1420 Multilabel Counter (PerkinElmer). Each assay was conducted in triplicate and repeated three times. Combination index (CI) was calculated using the CompuSyn program (ComboSyn Inc).

### Western blotting

Western blotting was performed using antibodies listed in Table S1 as previously described 16.

### Cellular thermal shift assay (CETSA)

Binding of Mcl-1 inhibitors to endogenous Mcl-1 was analyzed by CETSA based on a published protocol 17. Briefly, cells were treated with Mcl-1 inhibitors or control 0.1% DMSO for 2 hours in T-75 flasks. After treatment, cells were harvested, washed once with 1× PBS, resuspend in 750 μL HBSS, and lysed by 4 cycles of freezing (dry ice/ethanol for 5 min) and thawing (37 °C for 5 min). Samples were then distributed equally into 0.2-mL PCR tubes and heated at different temperatures for 3 min on a thermal cycler (Hybaid), followed by centrifugation at 13,200× rpm for 5 min and analysis of supernatants by western blotting.

### Immunoprecipitation (IP)

Cell lysis, preparation of cell lysates, and IP using 1-2 µg of IP antibodies (Table S1) was performed as previously described 18. The precipitates suspended in 2× Laemmli sample buffer were analyzed by SDS-PAGE and western blotting.

### Analysis of apoptosis

Adherent and floating cells were harvested after treatment. Apoptosis was measured by counting condensed and fragmented nuclei after nuclear staining with Hoechst 33258 (Invitrogen) as previously described 19. At least 300 cells were analyzed for each sample. Annexin V/propidium iodide (PI) staining was performed using Annexin-Alexa Fluor 488 (Invitrogen) and PI as described 6. Colony formation assays were performed by plating treated cells in 6-well plates at appropriate dilutions, followed by crystal violet staining 14 days later as described 6. Each experiment was performed in triplicate and repeated at least twice.

### Genomic PCR and sequencing

To verify *Mcl-1*-KI cell lines and detect *FBW7* hotspot mutations in regorafenib-resistant CRC cells and patient-derived samples, genomic DNA was isolated by using ZR-96 Quick-gDNA Kit (ZYMO Research) according to the manufacturer's instructions. One μL out of 50 μL genomic DNA preparation was amplified by PCR using previously described cycle conditions 9 and primer pairs for: *Mcl-1* KI: 5'-GGGTCTTCCCCAGTTTTCTC-3'/5'-AATGAACCCCCTTACCTTGG-3'; *FBW7* R465: 5'-CCCAACTTCCCATTCCCTTA-3'/5'-ATTAGTATGCCCCTGCAACG-3'; and *FBW7* R479/R505: 5'-GGTGGAGTATGGTCATCACAAA-3'/5'-CAAAACGCTATGGCTTTCCT-3'.

### Analysis of patient-derived CRC organoids

Patient-derived CRC organoids were established using surgically resected CRC tissues from the Pitt Biospecimen Core (PBC) at University of Pittsburgh as described 20. Tissues were acquired with informed consent and approval by the University of Pittsburgh Ethics Committee. CRC organoids were cultured in Matrigel (Corning) incubated with advanced DMEM/F12 (Invitrogen) medium with supplements (Table S1), including 50% (v/v) L-WRN-conditioned medium containing Wnt3a, R spondin , and Noggin prepared as described 20, 1× penicillin/streptomycin (Invitrogen), 10 mM HEPES (Invitrogen), 2 mM GlutaMAX (Invitrogen), 1× B27 (Invitrogen), 1× N2 (Invitrogen), 1 mM N-Acetylcysteine (Sigma), 10 nM [leu-15]-Gastrin (Sigma), 10 mM nicotinamide (Sigma), 10 μM SB202190 (Sigma), 50 ng/mL recombinant murine EGF (Peprotech), and 0.5 μM A83-01 (Tocris Bioscience).

Before treatment, organoids were digested into small clumps and seeded into 24-well or 96-well plates at appropriate density and cultured for 2 days. After treatment, organoid cell viability was analyzed by using the CellTiter-Glo® 3D Cell Viability Assay Kit (Promega) according to the manufacture's protocol. Active caspase 3 in organoids was analyzed by immunostaining as described 21. Quantitation of active caspase 3 was analyzed by using SensoLyte ® Homogeneous AMC Caspase-3/7 Assay Kit (AnaSpec). Results were obtained from at least three independent experiments with triplicate wells in each experiment.

### Animal experiments

All animal experiments were approved by the University of Pittsburgh Institutional Animal Care and Use Committee. Mice were housed in a sterile environment with micro isolator cages and allowed access to water and chow *ad libitum*. Cell line xenografts were established by subcutaneously injecting 4×10^6^ HCT116, *Mcl-1*-KI, or HCT116-R cells into both flanks of 5-6-week-old female Nu/Nu mice (Charles River). Syngeneic tumors were established by injecting 5×10^5^ CT26 cells in to into both flanks of 5-6-week-old BABL/cJ mice (Jackson Laboratory). PDX tumors were established and propagated in 5-6-week-old female NOD.Cg-Prkdcscid Il2rgtm1Wjl/SzJ (NSG) mice (Jackson Laboratory) as described 22. PDX1 was established from a *FBW7*-mutant and microsatellite stable (MSS) tumor (T4N0M1) in the sigmoid colon of a 77-year-old male. PDX2 was established from a *FBW7-*WT and MSS tumor (T2N0) in the right colon of a 69-year-old male. Xenograft tumors reached ~60 mm^3^ in size before treatment.

Tumor-bearing mice were randomized into different groups and treated with regorafenib (oral gavage; 20 mg/kg) with or without a combination with S63845 (i.p.; 20 mg/kg). S63845 was dissolved in 25 mM HCl, 20% hydroxypropyl-β-cyclodextrin (Sigma). Regorafenib was dissolved in Cremephor EL/95% ethanol (50:50) as a 4× stock solution and diluted to the final concentration with sterile water before use. Six mice were included in each group. Tumor growth was monitored by calipers, and tumor volumes were calculated according to the formula 1/2×length×width^2^. Ethical endpoint was defined as a time point when a tumor reached 1.5 cm or more in any dimension.

Tumor tissues were dissected and fixed in 10% formalin and embedded in paraffin. Terminal deoxynucleotidyl transferase mediated dUTP Nick End Labeling (TUNEL; EMD Millipore) and active caspase 3 (Cell Signaling Technology) immunostaining was performed on 5 μm paraffin-embedded tumor sections as described 9. Signals were detected by using AlexaFluor 488-conjugated secondary antibody (Invitrogen) with nuclear counter staining by 4'6-Diamidino-2-phenylindole (DAPI).

### Statistical Analysis

Statistical analyses were performed using GraphPad Prism Ⅵ software. *P* values were calculated by the Student t test between two groups or one-way ANOVA in three or more groups and considered significant if *P*< 0.05. The mean ± s.d. was displayed in the figures.

## Results

### Mcl-1 inhibitors restore regorafenib sensitivity in CRC cells expressing non-degradable Mcl-1

**Figure 1:**
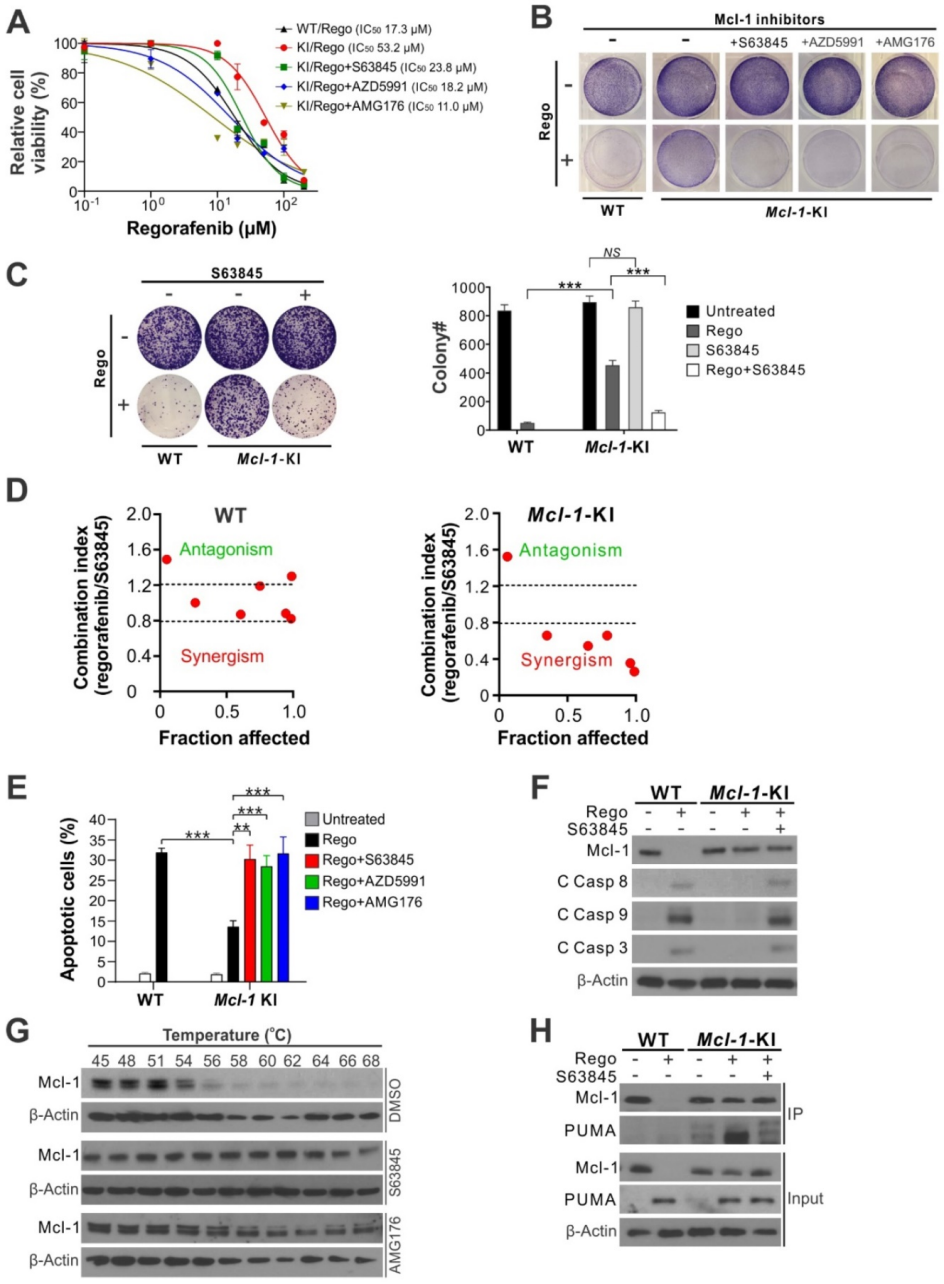
** Mcl-1 inhibitors restores apoptosis response and sensitivity to regorafenib in *Mcl***-***1*-KI HCT116 cells.** (**A**) MTS analysis of wild-type (WT) and *Mcl*-*1*-KI HCT116 cells treated with regorafenib at indicated concentrations with or without a combination with an indicated Mcl-1 inhibitor (5 μM) for 72 hours. (**B**) Crystal Violet staining of WT and *Mcl*-*1*-KI HCT116 cells treated with regorafenib (40 μM), an indicated Mcl-1 inhibitor (5 μM), or their combination for 48 hours. (**C**) Colony formation of WT and *Mcl*-*1*-KI HCT116 cells treated with regorafenib (40 μM), S63845 (5 μM), or their combination for 48 hours. *Left panel*: representative pictures of colonies visualized by crystal violet staining 2 weeks after treatment; *right panel*: enumeration of colony numbers. (**D**) Combination index (CI) and fraction affected of regorafenib and S63845 combining at different concentrations in WT and *Mcl*-*1*-KI HCT116 cells treated for 48 hours (shown in Figure S1B-C) were analyzed by the CompuSyn program (ComboSyn). **(E)** Apoptosis in cells treated as in **B** was analyzed by counting condensed and fragment nuclei after nuclear staining with Hoechst 33258. (**F**) Western blotting of Mcl-1 and cleaved (C) caspases 3, 8 and 9 in cells treated as in **C**. (**G**) Cellular thermal shift assay (CETSA) for the binding of indicated inhibitors to endogenous Mcl-1 in HCT116 cells. Cells were treated with the control DMSO or an indicated Mcl-1 inhibitor (5 μM) for 2 hours, followed by heating of cell lysates at indicated temperatures for 3 minutes and probing of supernatants by Mcl-1 western blotting. (**H**) Immunoprecipitation (IP) analysis of the binding between endogenous Mcl-1 and PUMA in cells treated as in** C** for 24 hours. In **A**, **C** and **E**, results were expressed as means ± s.d. of three independent experiments. **, *P* <0.01; ***, *P* <0.001.

Our previous study showed that knock-in (KI) of an Mcl-1 phosphorylation site mutant (S121A/E125A/S159A/T163A) in regorafenib-sensitive HCT116 CRC cells (Figure S1A) blocks regorafenib-induced Mcl-1 degradation and subsequent apoptotic response 9. We tested whether small-molecule Mcl-1 inhibitors can restore regorafenib sensitivity in an Mcl-1-specific manner in isogenic *Mcl-1*-KI cells relative to the parental HCT116 cells. Treating *Mcl-1*-KI cells with the Mcl-1 inhibitor S63845, AZD5991, or AMG176 completely restored regorafenib sensitivity in *Mcl-1*-KI cells, indicated by regorafenib inhibitory concentration 50 (IC_50_) (Figure 1A), crystal violet staining of viable cells (Figure 1B), and long-term cell survival assayed by colony formation (Figure 1C). A marked synergism between regorafenib and S63845, as indicated by CI (combination index) < 0.8, was observed in *Mcl-1*-KI cells, but not in WT HCT116 cells (Figure 1D and Figure S1B-C). Mcl-1 inhibitors also fully restored regorafenib-induced apoptosis, as determined by nuclear fragmentation (Figure 1E), annexin V staining (Figure S1D), and activation of caspases 3, 8 and 9 (Figure 1F). Analysis of different Bcl-2 family proteins showed that PUMA induction and Mcl-1 degradation are critical events in regorafenib-induced apoptosis in CRC cells (Figure S2A) 9, 23. These results demonstrate that regorafenib resistance is mediated by Mcl-1 in the *Mcl-1*-KI model, and that pharmacologic inhibition of Mcl-1 can restore sensitivity to regorafenib.

To further investigate the biochemical activity of the Mcl-1 inhibitors S63845 and AMG176, we utilized a cellular thermal shift assay (CETSA) on the parental HCT116 cells. This analysis showed that treatment with S63845 or AMG176 markedly protected endogenous Mcl-1 from heat-induced denaturation, indicating strong binding of these inhibitors to Mcl-1 (Figure 1G). Immunoprecipitation of Mcl-1 protein demonstrated strong binding of Mcl-1 to PUMA in regorafenib-treated *Mcl-1*-KI cells. However, this binding was disrupted by treatment with S63845 (Figure 1H). PUMA is a proapoptotic BH3-only Bcl-2 family protein required for regorafenib-induced apoptosis 23. Consistent with the results from HCT116 cells (Figure 1), Mcl-1 inhibitors alone do not affect viability or induce apoptosis in different CRC cell lines (Figure S2B) 9. These results demonstrate that the Mcl-1 inhibitors can overcome Mcl-1-mediated regorafenib resistance by relieving its inhibition on proapoptotic proteins such as PUMA.

### Mcl-1 inhibitors re-sensitize *FBW7*-mutant CRC cells to regorafenib by restoring apoptotic response

**Figure 2:**
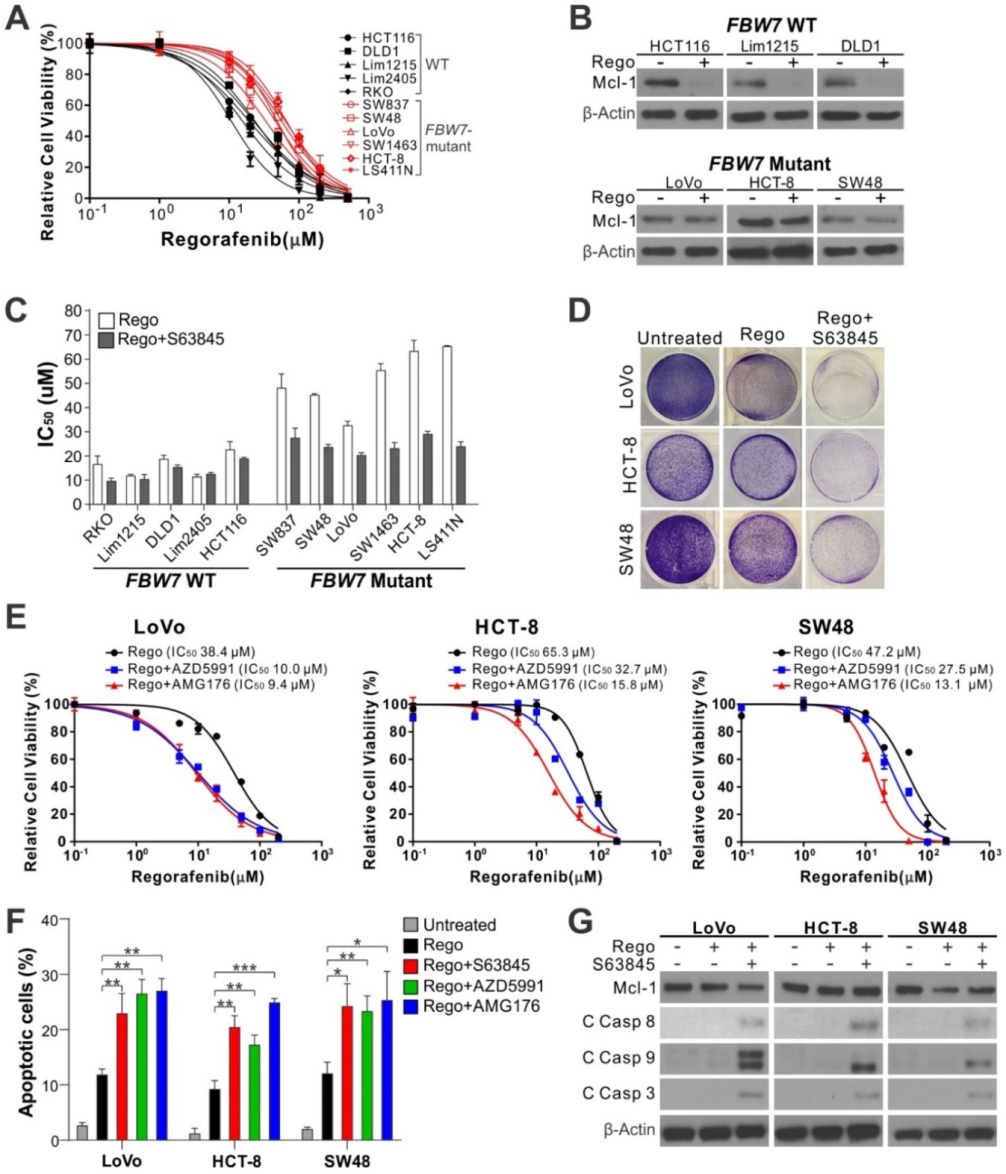
** Mcl-1 inhibitors re-sensitize *FBW7***-**mutant CRC cells to regorafenib.** (**A**) MTS analysis of indicated WT (black) and *FBW7-*mutant (red) CRC cell lines treated with regorafenib at indicated concentrations for 72 hours. (**B**) Western blotting of Mcl-1 in indicated WT and -mutant CRC cell lines treated with regorafenib (40 μM) for 8 hours. (**C**) Comparison of regorafenib IC_50_ in indicated WT and *FBW7*-mutant CRC cell lines treated with regorafenib alone or in combination with S63845 (5 μM) for 72 hours. (**D**) Crystal violet staining of *FBW7*-mutant LoVo, HCT-8, and SW48 cells treated with regorafenib (40 μM) alone or in combination with S63845 (5 μM) for 48 hours. (**E**) MTS analysis of *FBW7*-mutant LoVo, HCT-8, and SW48 cells treated with regorafenib at indicated concentrations alone or in combination with an indicated Mcl-1 inhibitor (5 μM) for 72 hours. (**F**) Apoptosis in cells treated as in **D** and **E** for 48 hours was analyzed by counting condensed and fragment nuclei after nuclear staining. (**G**) Western blotting of cleaved (C) caspases 3, 8 and 9 in cells treated as in **D**. In **A**, **C**, **E**, and **F**, results were expressed as means ± s.d. of three independent experiments. *,* P* < 0.05; **, *P* <0.01; ***, *P* <0.001.

*FBW7*, a tumor suppressor and E3 ubiquitin ligase of Mcl-1, is frequently mutated in CRCs, which underlies intrinsic regorafenib resistance in CRC cells 6. Compared to *FBW7*-WT cells, *FBW7*-mutant CRC cells were much more refractory to regorafenib (Figure 2A and Figure S3A), and deficient in regorafenib-induced Mcl-1 degradation and apoptosis (Figure 2B and Figure S3B) 6. Combining regorafenib with any Mcl-1 inhibitor markedly lowered regorafenib IC_50_ and restored regorafenib-induced loss of cell viability, apoptosis, and caspase activation in *FBW7*-mutant CRC cell lines 6, including SW837, SW48, LoVo, SW1463, HCT-8, and LS411N (Figure 2C-G and Figure S3C-D). In contrast, Mcl-1 inhibition had virtually no effect on regorafenib sensitivity and apoptosis in WT cell lines 6, including RKO, HCT116, DLD1, Lim1215, and Lim2405 (Figure 2C and Figure S3C-D). Similar observations were made in *FBW7*-WT and -mutant cells treated with sorafenib (Figure S3E-F), an analog of regorafenib approved for treating liver cancer and other gastrointestinal malignancies 24.

To verify if *FBW7* status is a key factor in determining the response to Mcl-1 inhibition in CRC cells, we analyzed isogenic *FBW7*-knockout (KO) HCT116 cells generated by homologous recombination 7. Similar to *FBW7*-mutant cells, *FBW7*-KO cells were much less sensitive to regorafenib and deficient in regorafenib-induced apoptosis and caspase activation (Figure S4A-E). Mcl-1 inhibition by S63845, AZD5991, or AMG176 restored regorafenib sensitivity, apoptosis, and caspase activation in *FBW7*-KO cells (Figure S4A-E). Similar observations were also made on *FBW7*-KO cells treated with sorafenib (Figure S4F). Therefore, the results from both *FBW7*-mutant and *FBW7*-KO CRC cells demonstrate that Mcl-1 inhibitors can overcome regorafenib resistance in *FBW7*-deficient CRC cells.

### Mcl-1 inhibitors overcome acquired resistance to regorafenib in CRC cells by restoring apoptotic response

**Figure 3:**
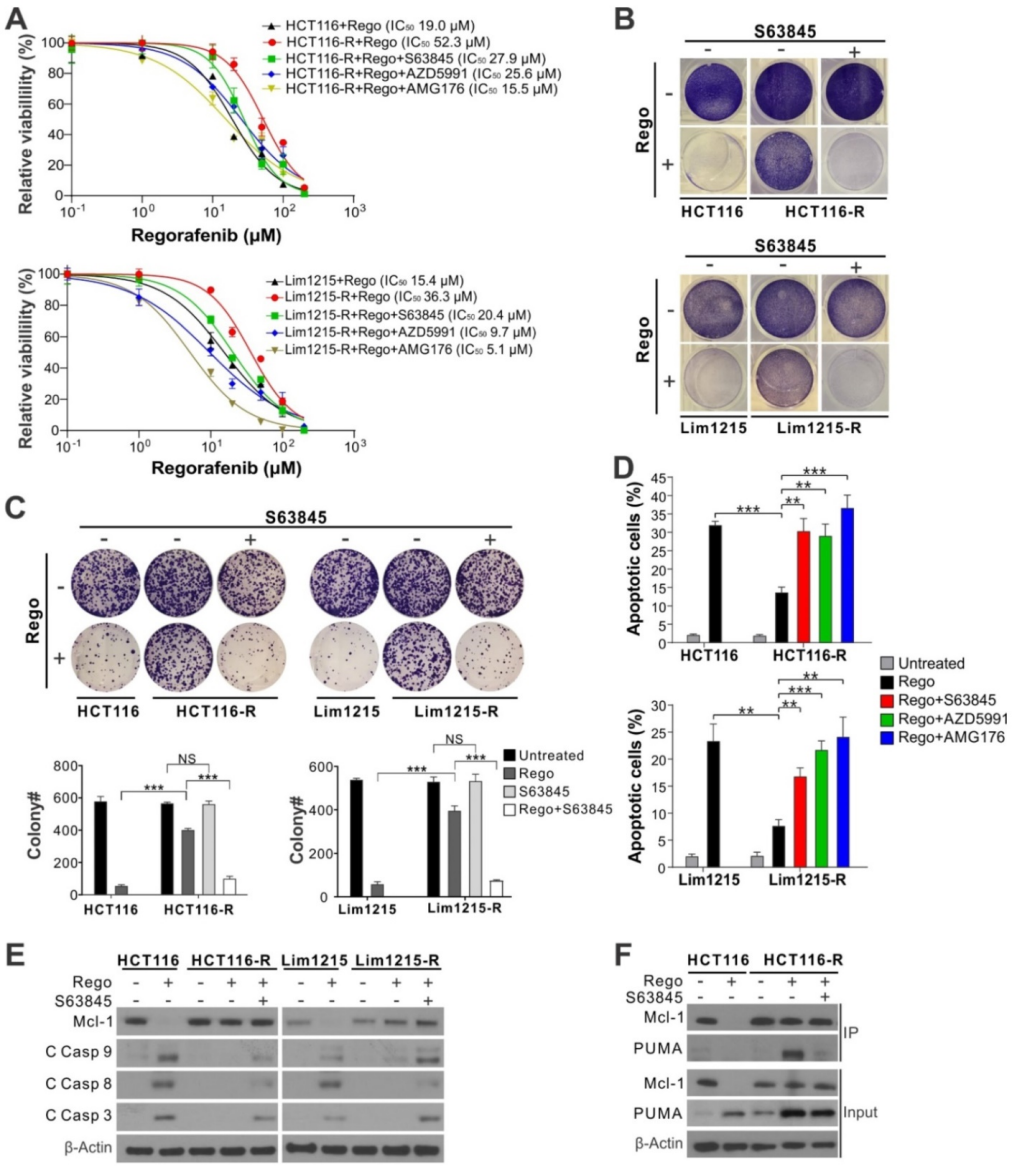
** Mcl-1 inhibitors re-sensitize CRC cells with acquired resistance to regorafenib.** (**A**) MTS analysis of parental and regorafenib-resistant HCT116 (HCT116-R) and Lim1215 (Lim1215-R) cells treated with regorafenib at indicated concentrations alone or in combination with an indicated Mcl-1 inhibitor (5 μM) for 72 hours. (**B**) Crystal violet staining of parental and regorafenib-resistant HCT116 and Lim1215 cells treated with regorafenib (40 μM) alone or in combination with S63845 (5 μM) for 48 hours. (**C**) Colony formation of cells treated as in (B). *Upper panel*: representative pictures of colonies visualized by crystal violet staining 2 weeks after treatment; *lower panel*: enumeration of colony numbers. (**D**) Apoptosis in cells treated with regorafenib (40 μM) alone or in combination with indicated Mcl-1 inhibitor (5 μM) for 48 hours was analyzed by counting condensed and fragment nuclei. (**E**) Western blotting of Mcl-1 and cleaved (C) caspases 3, 8, and 9 in indicated cells treated as in **C**. (**F**) IP analysis of the binding between endogenous Mcl-1 and PUMA in parental HCT116 and HCT116-R cells treated as in **C** for 24 hours. In **A**, **C** and **D**, results were expressed as means ± s.d. of three independent experiments. *NS*, not significant, *P* >0.05; **, *P* <0.01; ***, *P* <0.001.

Our previous study showed that regorafenib-resistant CRC cells established by multiple rounds of drug selection are deficient in Mcl-1 degradation and often enriched in *FBW7* hotspot mutations 6. We tested if Mcl-1 inhibitors can be used to restore regorafenib sensitivity in regorafenib-resistant HCT116 (HCT116-R) and Lim1215 (Lim1215-R) cells, which contain enriched *FBW7* R505C and R465C hotspot mutations, respectively 6. Treating HCT116-R and Lim1215-R cells with regorafenib combined with S63845, AZD5991 or AMG176 completely restored regorafenib sensitivity relative to the parental HCT116 and Lim1215 cells, as shown by decreased IC_50_, loss of cell viability, and suppression of colony formation (Figure 3A-C). Induction of apoptosis and caspase activation were also restored (Figure 3D-E and Figure S4G), as well as the dissociation of Mcl-1 and PUMA (Figure 3F). These data indicate that Mcl-1 inhibition can overcome acquired regorafenib resistance by liberating PUMA from Mcl-1 and subsequently restoring apoptosis.

### Mcl-1 inhibition overcomes regorafenib resistance in xenograft tumors

**Figure 4:**
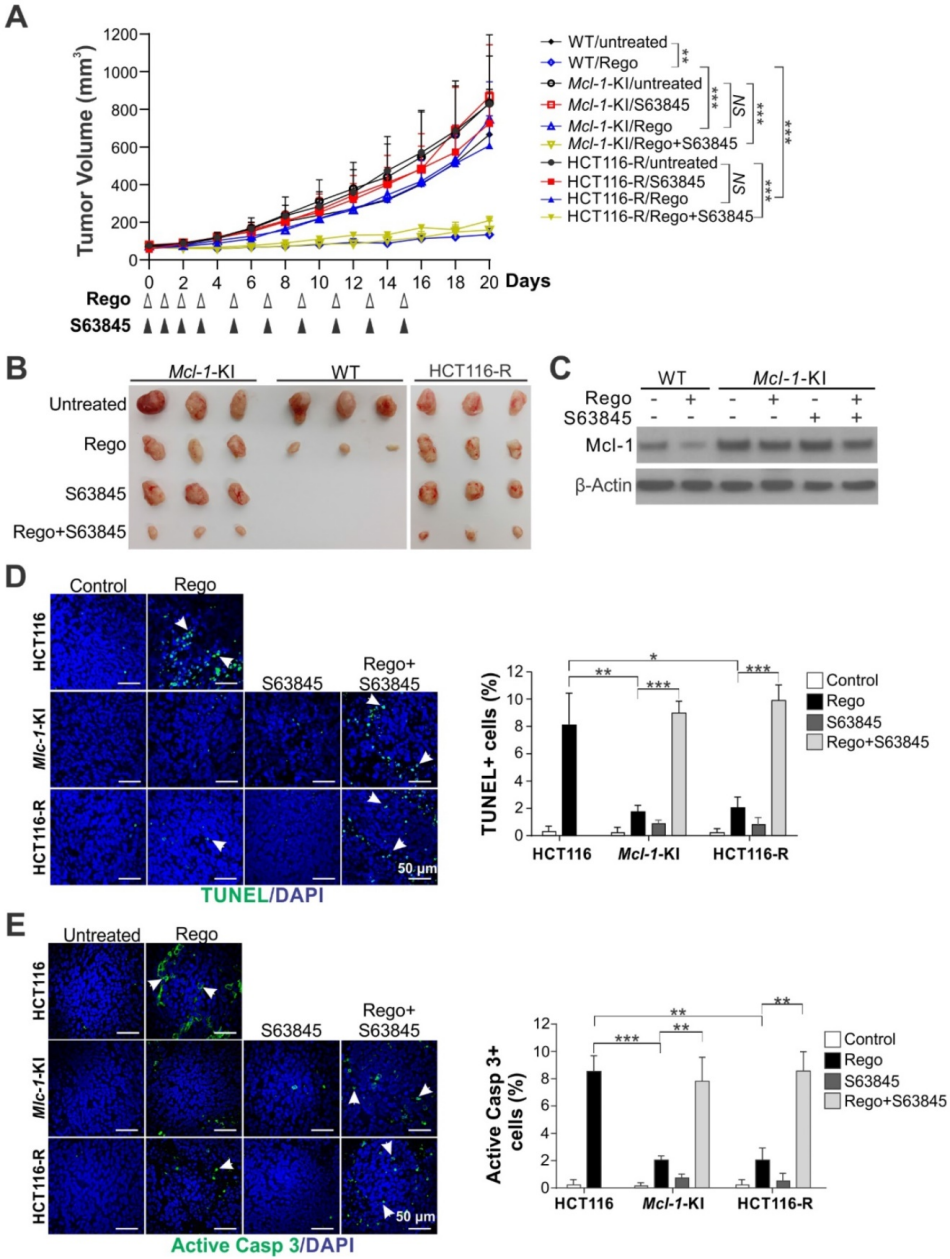
** Mcl-1 inhibition overcomes intrinsic and acquired resistance to regorafenib in xenograft tumors.** (**A**) Nude mice were injected s.c. with 4 × 10^6^ parental HCT116, *Mcl-1*-KI or HCT116-R cells. After tumor volume reached ~60 mm^3^, mice were treated with regorafenib (oral gavage; 20 mg/kg) alone or in combination with S63845 (i.p.; 20 mg/kg) as indicated. Tumor volume at indicated time points after treatment was calculated and plotted (n=6 in each group). (**B**) Representative pictures of tumors at the end of the experiment in **A**. (**C**) Xenograft tumors established and treated as in **A** for 4 consecutive days were randomly selected and analyzed for Mcl-1 by Western blotting. (**D**) and (**E**), Paraffin-embedded sections of tumor tissues from **C** were analyzed by (**D**) TUNEL and (**E**) active caspase 3 staining. *Left*, representative staining pictures with nuclear counterstaining by DAPI; *right*, quantification of positive cells. In **D** and **E**, arrows indicate example cells with positive staining. Results were expressed as means ± s.d. of three independent experiments. *,* P* < 0.05; **, *P* <0.01; ***, *P* <0.001.

To determine if Mcl-1 inhibition can reverse intrinsic and acquired resistance to regorafenib *in vivo*, we compared the effects of regorafenib alone or in combination with S63845 on parental HCT116, *Mcl-1*-KI, and HCT116-R xenograft tumors established in nude mice. Regorafenib (oral gavage; 20 mg/kg) alone significantly suppressed the growth of the parental HCT116 tumors and induced Mcl-1 degradation; but had little effect on *Mcl-1*-KI and HCT116-R tumors (Figure 4A-C). S63845 treatment (IP; 20 mg/kg), though having no effect by itself, completely reversed regorafenib resistance in *Mcl-1*-KI and HCT116-R tumors when used in combination with regorafenib (Figure 4A-B). TUNEL and active caspase 3 immunostaining showed that S63845 fully restored regorafenib-induced apoptosis in *Mcl-1*-KI and HCT116-R tumors relative to the parental HCT116 tumors (Figure 4D-E). S63845 with or without regorafenib was well tolerated and did not significantly decrease animal weight (Figure S5A), or cause cell loss or histological changes in normal tissues from heart, liver, spleen, lung and kidney (Figure S5B). Furthermore, S63845 had no effect on *FBW7*-WT CT26 syngeneic tumors in BABL/cJ mice with or without regorafenib (Figure S6) 25. These results indicate that Mcl-1 inhibition can overcome *in vivo* resistance to regorafenib caused by Mcl-1 degradation deficiency.

### Mcl-1 inhibition sensitizes *FBW7*-mutant but not WT PDOs to regorafenib

**Figure 5:**
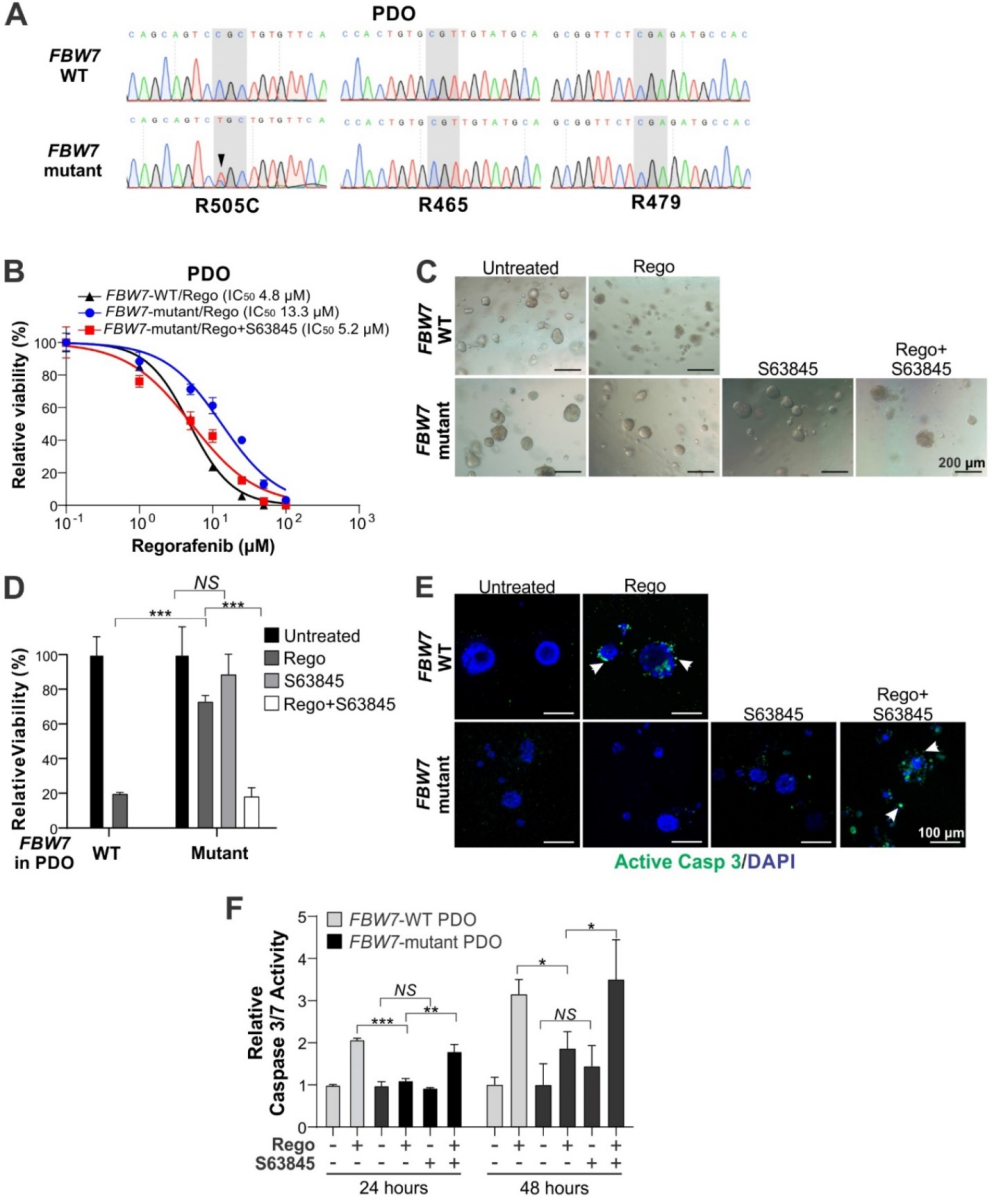
** Mcl-1 inhibition re-sensitizes *FBW7*-mutant PDO to regorafenib.** (**A**) DNA sequencing of the targeted genomic regions in CRC patient-derived organoids (PDOs) highlighting WT and corresponding *FBW7* mutant sequences. (**B**) Indicated CRC PDOs were treated with regorafenib at different concentrations alone or in combination with S63845 (1 μM) for 72 hours. Cell viability was assessed by 3D Cell Viability Assay Kit. (**C**) and (**D**), Representative images (**C**) and quantification (**D**) of CRC PDOs treated with regorafenib (20 μM) alone or in combination with S63845 (1 μM) for 48 hours. (**E**) CRC PDOs treated as in (C) were analyzed by active caspase 3 staining images. Representative images are shown with DAPI for nuclear counter staining. (**F**) CRC PDOs treated as in **C** for 24 or 48 hours were analyzed by Caspase 3/7 Activity Assay. Results in **B**, **D** and **F** were expressed as means ± s.d. of three independent experiments. *NS*, *P* > 0.05; *,* P* < 0.05; **, *P* <0.01; ***, *P* <0.001.

To explore the translational potential of Mcl-1 inhibitors in CRC, we tested if the regorafenib/S63845 combination is efficacious against patient-derived CRC samples containing *FBW7* mutations. We established a panel of patient-derived organoids (PDOs) using surgical specimens from 12 CRC patients. Upon targeted genomic sequencing for *FBW7* mutational hotspots including R505, R465 and R479, we identified a PDO with a heterozygous dominant R505C mutation (Figure 5A). This mutation was previously shown to mediate intrinsic and acquired regorafenib resistance in LoVo and HCT116-R cells, respectively, by blocking Mcl-1 degradation 6. Interestingly, this *FBW7*-mutant PDO was from a T4N0M1 tumor with *KRAS* G13D and *NRAS* G12D and MSS, a profile associated with lack of clinical response to EGFR-targeted therapy and anti-PD-1 immunotherapy 26, 27. Compared to the control *FBW7*-WT PDO from a different patient, this *FBW7*-mutant PDO was substantially less sensitive to regorafenib (IC_50_ 13.3 *vs*. 4.8 µM) (Figure 5B), and showed significantly less growth inhibition, loss of cell viability, and activation of caspase 3/7 (Figure 5C-F). Importantly, S63845 fully restored regorafenib sensitivity as well as the induction of cell death and caspase 3/7 activation in this *FBW7*-mutant PDO (Figure 5B-F). In contrast, S63845 had little effect on the *FBW7*-WT PDO with or without regorafenib (Figure S7A-B). These results suggest that PDOs may be useful for predicting the response of Mcl-1 inhibitor and regorafenib combination therapy in *FBW7* mutated CRCs.

### Mcl-1 inhibition re-sensitizes *FBW7*-mutant PDX to regorafenib

**Figure 6:**
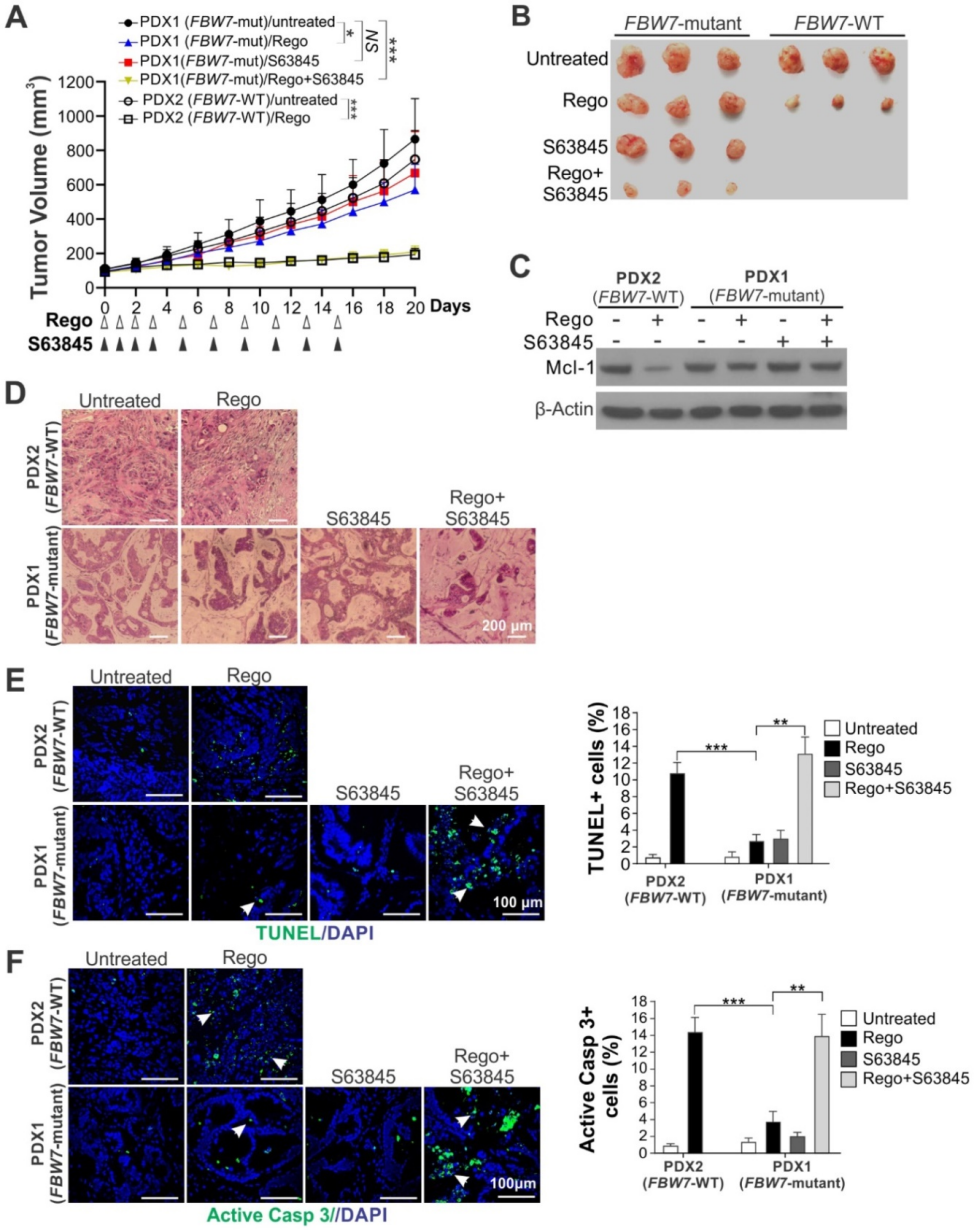
** Mcl-1 inhibition re-sensitizes *FBW7*-mutant PDX to regorafenib.** (**A**) NSG mice were subcutaneously implanted with PDX1 (*FBW7*-mutant) and PDX2 (*FBW7*-WT) tumors*.* After tumor volume reached ~60 mm^3^, mice were treated with regorafenib (oral gavage; 20 mg/kg) alone or in combination with S63845 (i.p.; 20 mg/kg) as indicated. Tumor volume at indicated time points after treatment was calculated and plotted (n=6 in each group). (**B**) Representative pictures of tumors at the end of the experiment in **A**. (**C**) PDX1 and PDX2 tumors established and treated as in **A** for 4 consecutive days were randomly selected and analyzed for Mcl-1 by Western blotting. (**D**)-(**F**), Paraffin-embedded sections of tumor tissues from **C** were analyzed by (**D**) H&E, (**E**) TUNEL, and (**F**) active caspase 3 staining. In **D** and **E**, arrows indicate example cells with positive staining. *Left*, representative staining pictures with nuclear counterstaining by DAPI; *right*, quantification of positive cells. Results were expressed as means ± s.d. of three independent experiments. *NS*, *P* > 0.05; *,* P* < 0.05; **, *P* <0.01; ***, *P* <0.001.

Compared with other *in vivo* models, PDX models better recapitulate heterogeneity, histology, and molecular alterations of patient tumors 28. We further explored the efficacy of the regorafenib/S63845 combination in a CRC PDX model with WT or mutant (R505C) *FBW7* (Figure S7C). Regorafenib was able to potently suppress the growth of the WT PDX, but caused significantly less growth suppression of the *FBW7*-mutant PDX (Figure 6A-B). Regorafenib was also able to induce Mcl-1 degradation in the WT PDX, but unable to induce a similar Mcl-1 degradation in the *FBW7*-mutant PDX (Figure 6C). S63845 completely reversed regorafenib resistance in the *FBW7*-mutant PDX (Figure 6A-B), and increased tumor cell loss as analyzed by H&E staining (Figure 6D), cell death by TUNEL staining (Figure 6E), and activation of caspase 3 (Figure 6F). No obvious toxicity or weight loss was observed in the NOD/SCID hosts with the treatment (Figure S7D). Collectively, our results demonstrate that Mcl-1 inhibitors can be used to develop a precision combination therapy for overcoming regorafenib resistance in CRCs.

## Discussion

Metastatic CRC is one of the most deadly cancers characterized by poor prognosis and low five-year survival rate of just 11% 29. Metastatic CRC patients are typically treated with conventional cytotoxic chemotherapy, targeted therapy, and more recently with anti-PD-1 immunotherapy 26. However, most CRCs are either inherently insensitive to therapeutic treatment or acquire resistance upon relapse 3. There is a critical need for developing novel and more effective CRC therapies, especially for those with intrinsic or acquired resistance to existing treatments 30.

Our results from isogenic cell line, xenograft, and patient-derived models demonstrate that inhibiting Mcl-1 is an effective approach for re-sensitizing CRCs with intrinsic and acquired resistance to regorafenib. Regorafenib is a multi-kinase inhibitor approved for treating CRC and other gastrointestinal malignancies 2, 31. The antitumor activity of regorafenib relies on degradation of the antiapoptotic protein Mcl-1 6. Aberrant Mcl-1 expression is frequently found in CRCs and significantly correlated with advanced tumor stages, lymph node metastasis, resistance to chemotherapy, and poor patient survival 32-35. Blocking Mcl-1 degradation abolished the response of CRC cells to a variety of anticancer agents, such as inhibitors of different kinases, heat shock proteins, and histone deacetylases 9, 18, 36. Targeting pro-survival Bcl-2 family proteins has led to recent approval of the Bcl-2-selective inhibitor Venetoclax 10. However, lack of Mcl-1 binding has limited the applications of Venetoclax and other Bcl-2 inhibitors, and Mcl-1 accumulation can cause resistance to these inhibitors 37, 38. Together, these studies provide compelling evidence that Mcl-1 is an attractive therapeutic target against CRC and other solid tumors, especially those that are refractory to other therapies.

The observed differences between WT and *FBW7*-mutant CRCs suggest that *FBW7* status is critical in determining the response to the regorafenib/Mcl-1 inhibitor combination. *FBW7* is frequently mutated in human cancers including 10-15% of CRCs 7, 8. *FBW7* mutations have a broad functional role in determining therapeutic responses of cancer cells 8, and can modulate responses to γ secretase inhibitors in leukemia cells 39, to histone deacetylase inhibitors in squamous tumor cells 40, and to antimitotic drugs in CRC cells 41. *FBW7* encodes an F-box protein that functions as a substrate receptor for SCF-type of ubiquitin ligase complexes 42. In addition to Mcl-1, FBW7 also mediates ubiquitination and degradation of other substrates, including Jun, Myc, cyclin E, and Notch 8. Due to the multi-functional nature of FBW7, the same mutation could produce highly variable outcomes depending on the tumor type and treatment type. Therefore, it is essential to understand the exact functional role of *FBW7* mutations to explore their use as a biomarker of therapeutic response.

The current study highlights multiple areas of translation. First, our results suggest that the presence of *FBW7* hotspot mutations can predict for poorer response to regorafenib. *FBW7* mutations may appear during the course of regorafenib treatment and indicate an acquired resistance mechanism, or they may be present in regorafenib-naïve patients and signal intrinsic resistance to regorafenib. Anticancer therapies including targeted therapies often generate highly heterogeneous patient responses and are associated with substantial toxicities. Hepatotoxicity and other side effects have been observed in regorafenib-treated patients 43. Using biomarkers to identify potential responders and stratify patients can prevent unnecessary treatments and avoid therapy-associated adverse effects. For example, *KRAS* and *BRAF* mutations have been routinely used to exclude anti-EGFR therapy in CRC patients 44, while MSI (microsatellite instable) predicts responsiveness of CRCs to anti-PD-1 immunotherapy 45. Second, the results from this study suggest that establishing and analyzing patient-derived tumor models, such as PDO and PDX models, can be used to screen tumors for identifying potential responders, and for designing personalized treatment. Finally, we have developed highly sensitive PCR assays that may be useful for monitoring responses and *FBW7* mutations in liquid biopsies 6.

In conclusion, we demonstrate that Mcl-1 inhibitors can be used to overcome mutant*-FBW7*-driven regorafenib resistance and help to develop a precision therapy for improving CRC treatment. The potential use of this strategy needs to be further assessed in clinical studies to determine the safety and efficacy of this combination, as well as the utility of *FBW7* mutations and Mcl‐1 level to predict therapeutic response.

## Supplementary Material

Supplementary figures and table.

## Abbreviations

CETSA: cellular thermal shift assay
CI: combination index
CRC: colorectal cancer
DAPI: 4' 6-Diamidino-2-phenylindole
DMSO: dimethylsulfoxide
FBW7: F-box and WD repeat domain-containing 7
IP: immunoprecipitation
KI: knock-in
KO: knockout
Mcl-1: myeloid cell leukemia 1
MSI: microsatellite instable
MSS: microsatellite stable
MTS: 3-(4,5-dimethylthiazol-2-yl)-5-(3-carboxymethoxyphenyl)-2-(4-sulfophenyl)-2H-tetrazolium
NSG: NOD.Cg-Prkdc^scid^ Il2rg^tm1Wjl^/SzJ
PDO: patient-derived organoid
PDX: patient-derived xenograft
PI: propidium iodide
RT-PCR: reverse transcriptase PCR
TUNEL: terminal deoxynucleotidyl transferase mediated dUTP nick end labeling
WT: wild-type
