# Supplementary material for: Mcl-1 inhibition overcomes intrinsic and acquired regorafenib resistance in colorectal cancer
Source: Theranostics. 2020 Jul 9;10(18):8098–110. doi: 10.7150/thno.45363 (PMC7381732; doi:10.7150/thno.45363)
Supplement: Supplementary file 1 — Supplementary figures and table. [file thnov10p8098s1.pdf]

Table S1. List of reagents

Reagents	Source	Catalogue #
**Anticancer agents and solvents**		
Regorafenib (for cell)	Active Biochem	#A-1486
Regorafenib (for animal)	LC Laboratories	#R-8024
Sorafenib	LC Laboratories	#S-8599
S63845	Fisher Scientific	#50-149-2689
AZD5991	WeiYuan Biotech	#A-6097
AGM176	WeiYuan Biotech	#A-6056
DMSO	Sigma-Aldrich	#D2650-5X
(2-Hydroxypropyl)-β-cyclodextrin	Cayman Chemical	#16169
Cremophor EL™	Fisher Scientific	#23-847-01SET
**Antibodies**		
Mouse monoclonal, Mcl-1	BD Biosciences	# 559027
Mouse monoclonal, β-Actin	Sigma-Aldrich	#A5441
Mouse monoclonal, cleaved caspase 8	Cell Signaling Technology	#9746
Rabbit polyclonal, cleaved caspase 3	Cell Signaling Technology	#9661
Rabbit polyclonal, cleaved caspase 9	Cell Signaling Technology	#9502
Rabbit polyclonal, PUMA	QCB	#3795
Rabbit polyclonal, Bid	Cell Signaling Technology	#2002
Rabbit polyclonal, Bcl-X_L	BD Biosciences	#610212
Rabbit polyclonal, Bim	Cell Signaling Technology	#2819
Mouse monoclonal, Bcl-2	Dako	# M0887
Mouse monoclonal, Noxa	EMD Millipore	# OP180
Goat anti-mouse secondary antibodies	Pierce	#31432
Goat anti-Rabbit secondary antibodies	Pierce	#31462
Goat anti-Rabbit secondary antibodies, AlexaFluor 488	Invitrogen	#A11012
**Kits**		
Tali™ Apoptosis Kit - Annexin V Alexa Fluor™ 488/Propidium Iodide	Invitrogen	#A10788
CellTiter-Glo® 3D Cell Viability Assay	Promega	#G9681
SensoLyte ® Homogeneous AMC Caspase - 3/7 Assay Kit	AnaSpec	#AS-71118
ApopTag Fluorescein In Situ Apoptosis Detection Kit (for TUNEL)	EMD Millipore	#S7110
CellTiter 96 AQueous One Solution Cell Proliferation Assay (MTS) Kit	Promega	#G3580
Zymoclean Gel DNA Recovery Kit	ZYMO Research	#D4002

Quick-gDNA Minipre Kit	ZYMO Research	#D3025
VectaShield + DAPI	Vector Laboratories	#H-1500
CellTiter-Glo 3D Cell Viability Assay	Promega	#G9681
**Reagents for organoid culture**		
L-WRN cells	ATCC	#CRL-3276
Matrigel matrix, Phenol-Red-free	Corning	#356231
Advanced DMEM/F-12	ThermoFisher	#12634010
Penicillin-Streptomycin (10,000 U/mL)	ThermoFisher	#15140122
HEPES (1 M)	ThermoFisher	#15630106
B-27 Supplement (50X), serum free	ThermoFisher	#17504044
N-2 Supplement (100X)	ThermoFisher	#17502048
N-acetylcysteine amide	Sigma	#A0737
GlutaMAX Supplement	ThermoFisher	#35050061
[Leu15]-Gastrin I human	Sigma	#G9145
Nicotinamide	Sigma	#N0636
SB 202190	Sigma	#S7067
Recombinant Murine EGF	Peprotech	#315-09
A 83-01	Tocris Bioscience	#2939

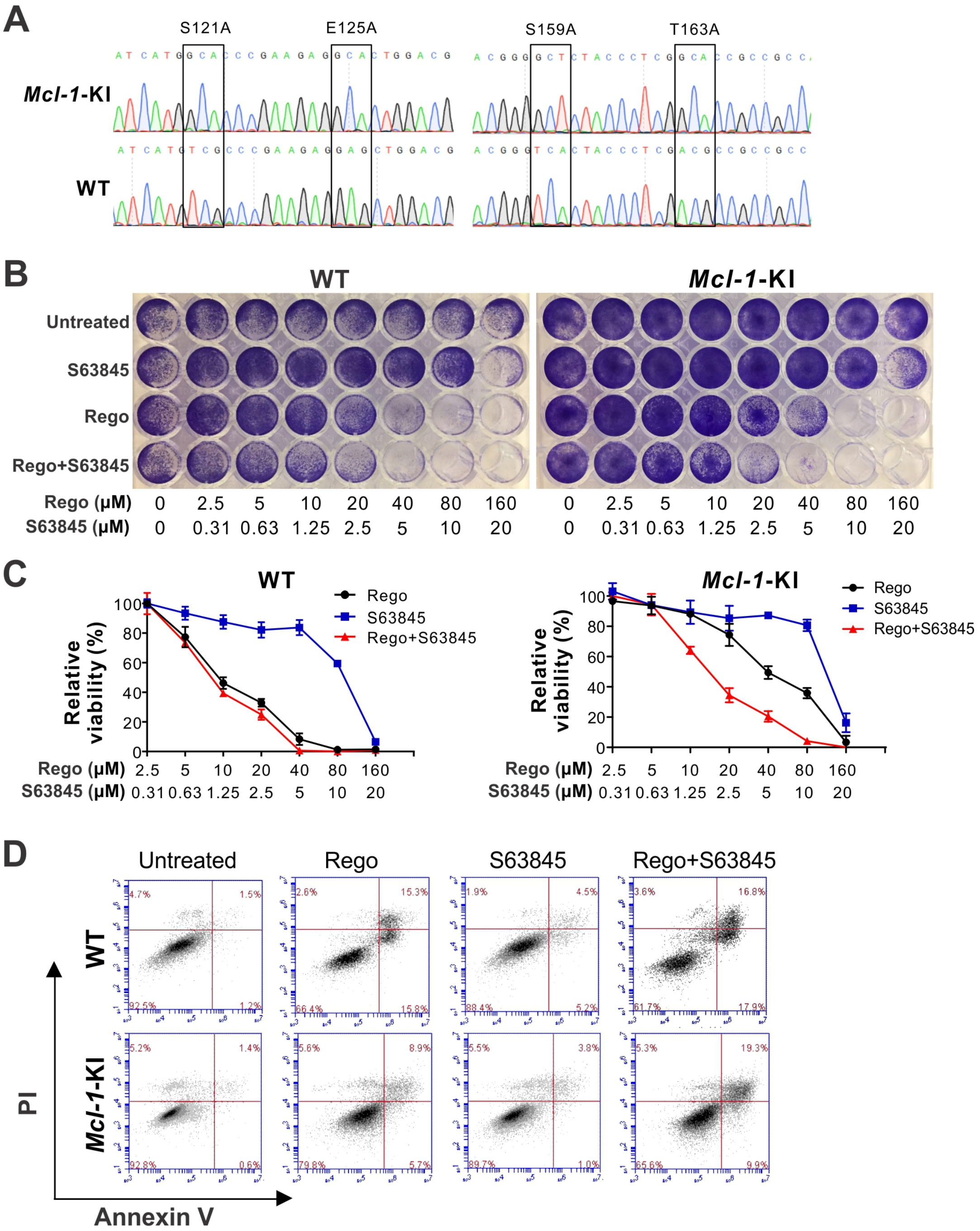

Figure S1. Mcl-1 inhibitors restore regorafenib sensitivity in *Mcl-1*-KI cells. (**A**) DNA sequencing of the targeted genomic regions in WT and *Mcl-1* knock-in (*Mcl-1*-KI) HCT116 cells highlighting WT and corresponding mutant sequences. (**B**) WT and *Mcl-1*-KI HCT116 cells treated with regorafenib combined with S63845 at indicated concentrations for 48 hours. Cell viability was analyzed by crystal violet staining and representative pictures are shown. (**C**) The results from **B** were quantified and expressed as means ± s.d. of three independent experiments. (**D**) Apoptosis in WT and *Mcl-1*-KI HCT116 cells treated with regorafenib (40 μM), S63845 (5 μM), or their combination for 48 hours was analyzed by annexin V/propidium iodide (PI) staining followed by flow cytometry. Signals in two right quadrants represent early (Annexin V+/PI-) and late (Annexin V+/PI+) apoptotic cells.

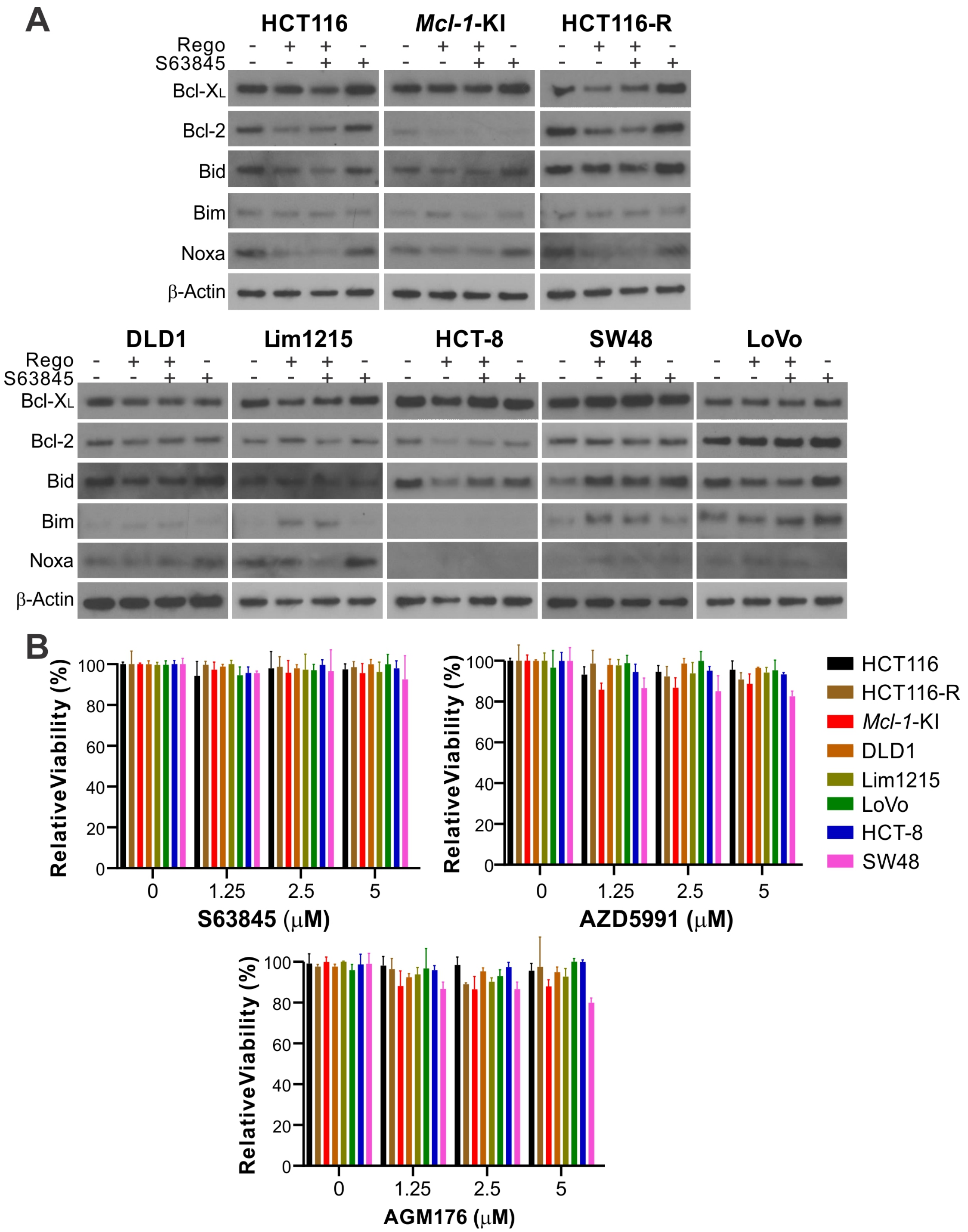

Figure S2. Effects of Mcl-1 inhibitors on the expression Bcl-2 family proteins and viability of CRC cells. Parental, *Mcl-1* knock-in (*Mcl-1*-KI) and regorafenib-resistant (HCT116-R) HCT116 cells, along with *FBW7*-WT DLD1 and Lim1215 and *FBW7*-mutant LoVo, HCT-8 and SW480 cells, were treated with regorafenib, an Mcl-1 inhibitor, or their combination. (**A**) Western blotting of indicated Bcl-2 family proteins in cells treated with regorafenib (40 μM), S63845 (5 μM), or their combination for 24 hours. (**B**) MTS analysis of viability of cells treated with different Mcl-1 inhibitors at indicated concentrations for 48 hours. Results were expressed as means ± s.d. of three independent experiments.

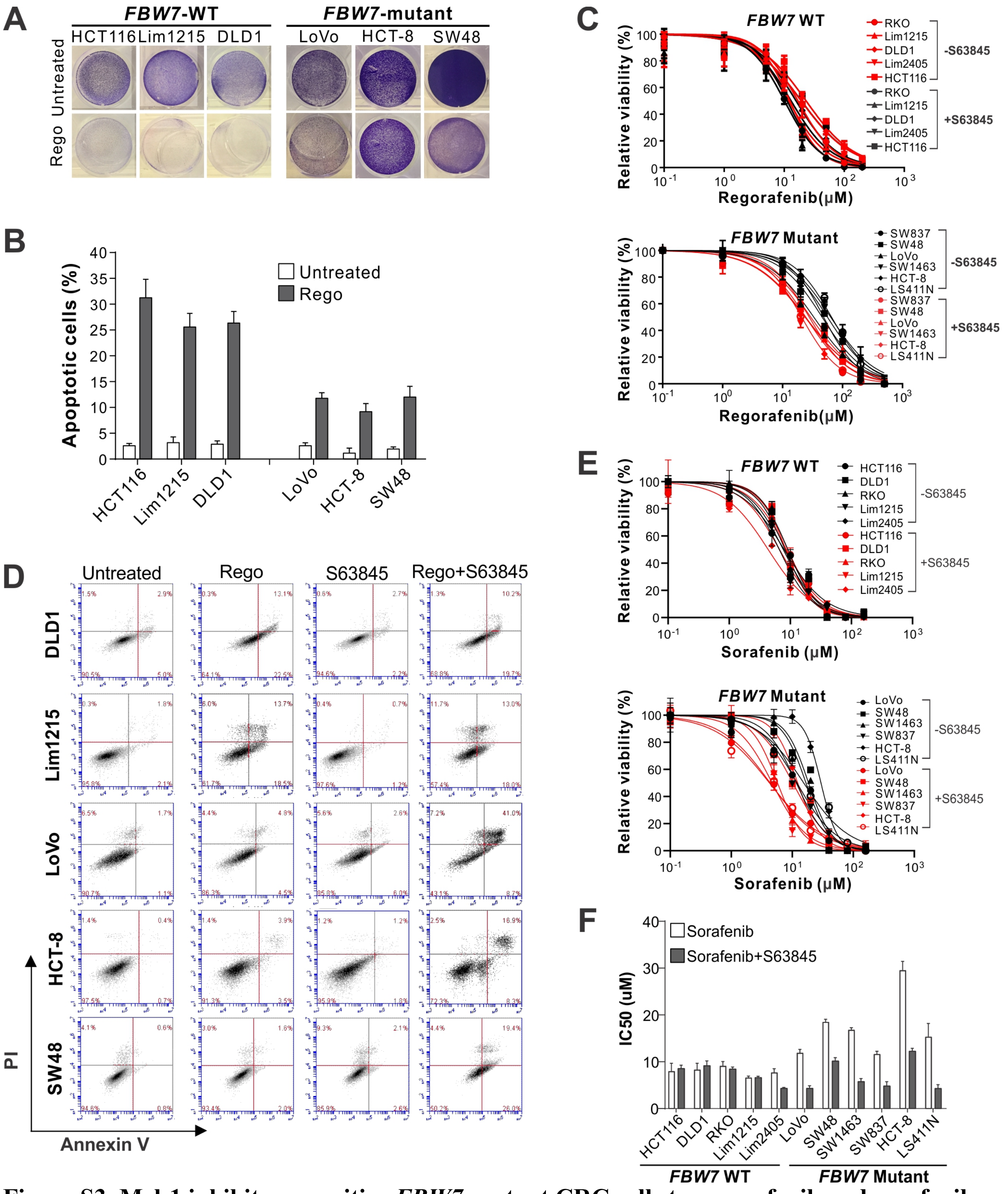

Figure S3. Mcl-1 inhibitors sensitize *FBW7*-mutant CRC cells to regorafenib and sorafenib. (**A**) Crystal violet staining of indicated WT and *FBW7*-mutant cell lines treated with regorafenib (40 μM) for 48 hours. (**B**) Apoptosis in cells treated as in **A** was analyzed by counting condensed and fragment nuclei after nuclear staining. (**C**) MTS analysis of indicated WT and *FBW7*-mutant CRC cell lines treated with regorafenib at indicated concentrations alone or in combination with S63845 (5 μM) for 72 hours. (**D**) Apoptosis in *FBW7*-WT Lim1215 and DLD1 cells and *FBW7*-

mutant LoVo, HCT-8, and SW48 cells treated with regorafenib (40 µM), S63845 (5 µM), or their combination for 48 hours was analyzed by annexin V/PI followed by flow cytometry. Signals in two right quadrants represent early (Annexin V+/PI-) and late (Annexin V+/PI+) apoptotic cells. (**E**) MTS analysis of indicated WT and *FBW7*-mutant CRC cell lines treated with sorafenib at indicated concentrations alone or in combination with S63845 (5 µM) for 72 hours. (**F**) Comparison of sorafenib IC_{50} in indicated WT and *FBW7*-mutant CRC cell lines treated with sorafenib alone or in combination with S63845 (5 µM) for 72 hours. Results in **B**, **C**, **E** and **F** were expressed as means ± s.d. of three independent experiments.

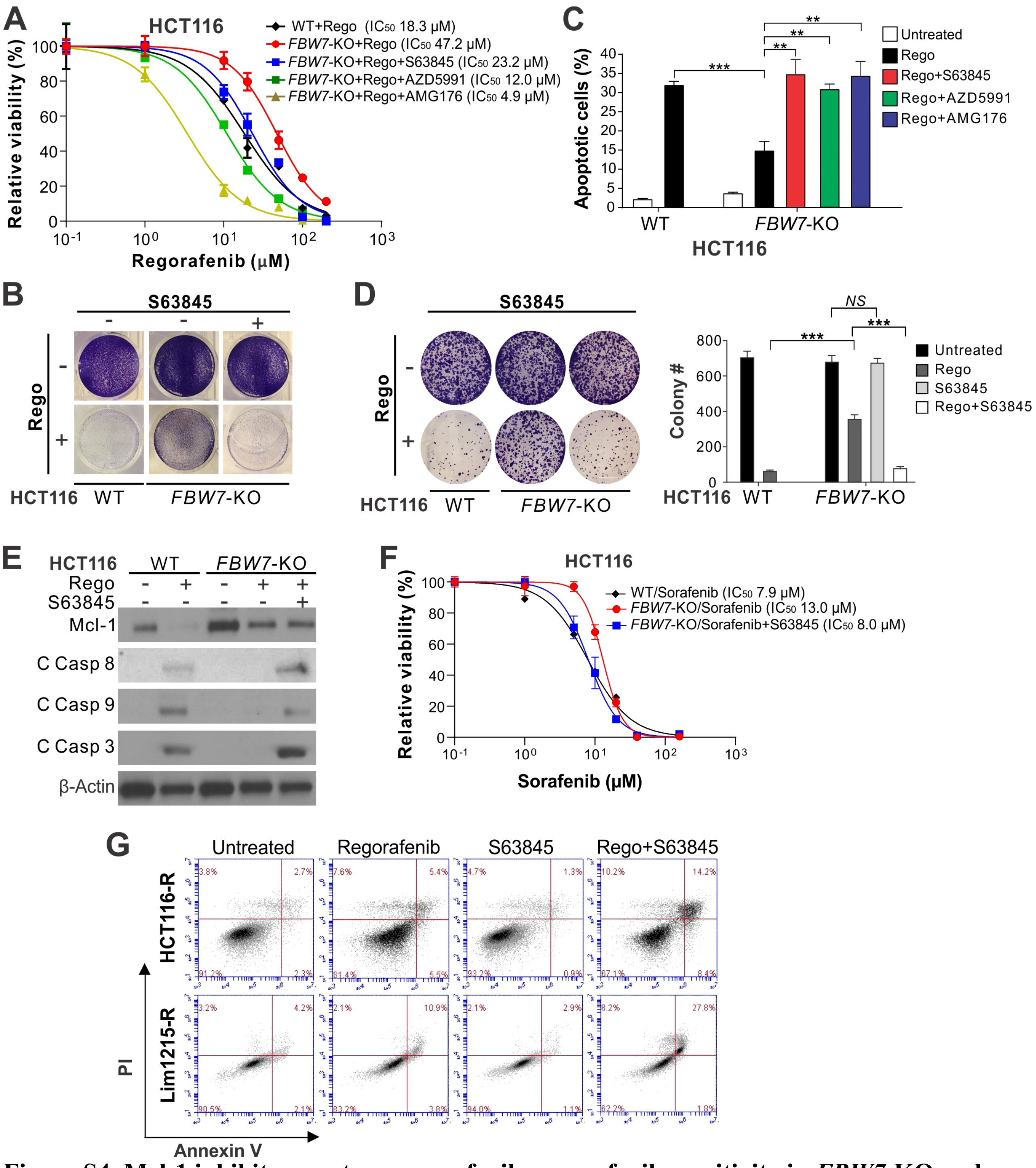

Figure S4. Mcl-1 inhibitors restore regorafenib or sorafenib sensitivity in *FBW7*-KO and regorafenib-resistant CRC cells. (**A**) MTS analysis of WT and *FBW7*-KO HCT116 cells treated with regorafenib at indicated concentrations alone or in combination with an indicated Mcl-1 inhibitor (5 μM) for 72 hours. (**B**) Crystal violet staining of WT and *FBW7*-KO HCT116 cells treated with regorafenib (40 μM) alone or in combination with S63845 (5 μM) for 48 hours. (**C**) Apoptosis in cells treated as in **A** and **B** for 48 hours was analyzed by counting condensed and fragment nuclei after nuclear staining. (**D**) Colony formation of cells treated as in **B**. *Upper panel*: representative pictures of colonies visualized by crystal violet staining 2 weeks after treatment;

lower panel: enumeration of colony numbers. (**E**) Western blotting of cleaved (C) caspases 3, 8, and 9 in cells treated as in **B**. (**F**) MTS analysis of indicated WT and *FBW7*-KO HCT116 cells treated with sorafenib at indicated concentrations alone or in combination with S63845 (5 μM) for 72 hours. (**G**) Apoptosis in regorafenib-resistant HCT116 (HCT116-R) and Lim1215 (Lim1215-R) cells treated with regorafenib (40 μM), S63845 (5 μM), or their combination for 48 hours was analyzed by annexin V/PI followed by flow cytometry. Signals in two right quadrants represent early (Annexin V+/PI-) and late (Annexin V+/PI+) apoptotic cells. Results in **A**, **C**, **D** and **F** were expressed as means ± s.d. of three independent experiments. **, P <0.01; ***, P <0.001.

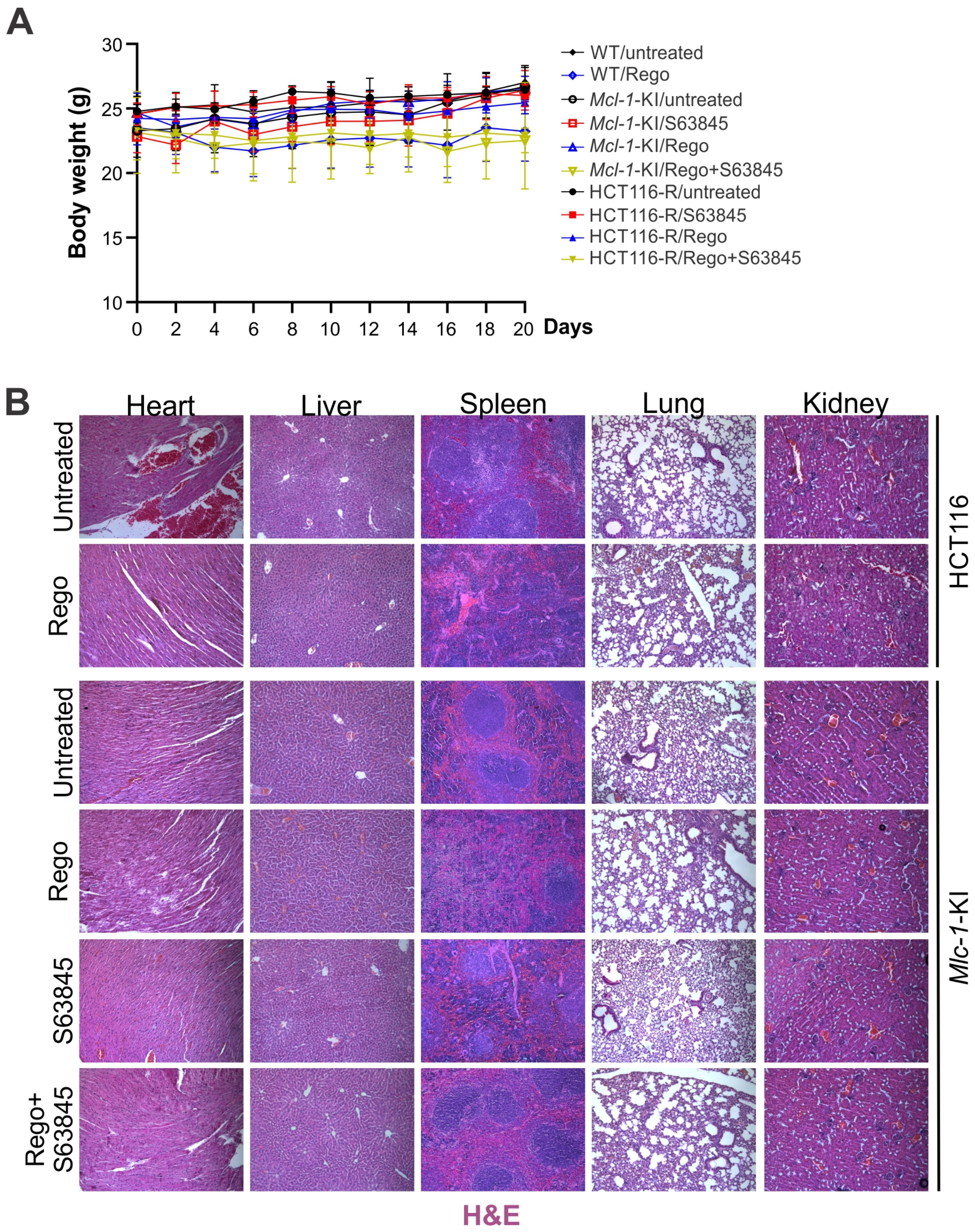

Figure S5. The regorafenib/S63845 combination is well tolerated in nude mice. (**A**) Body weight of nude mice harboring indicated xenograft tumors (n=6) at indicated time points after treatment with regorafenib (oral gavage; 20 mg/kg) alone or in combination with S63845 (i.p.; 20 mg/kg). (**B**) H&E staining of the indicated tissues from randomly selected mice harboring indicated xenograft tumors at the end of experiments in **A**.

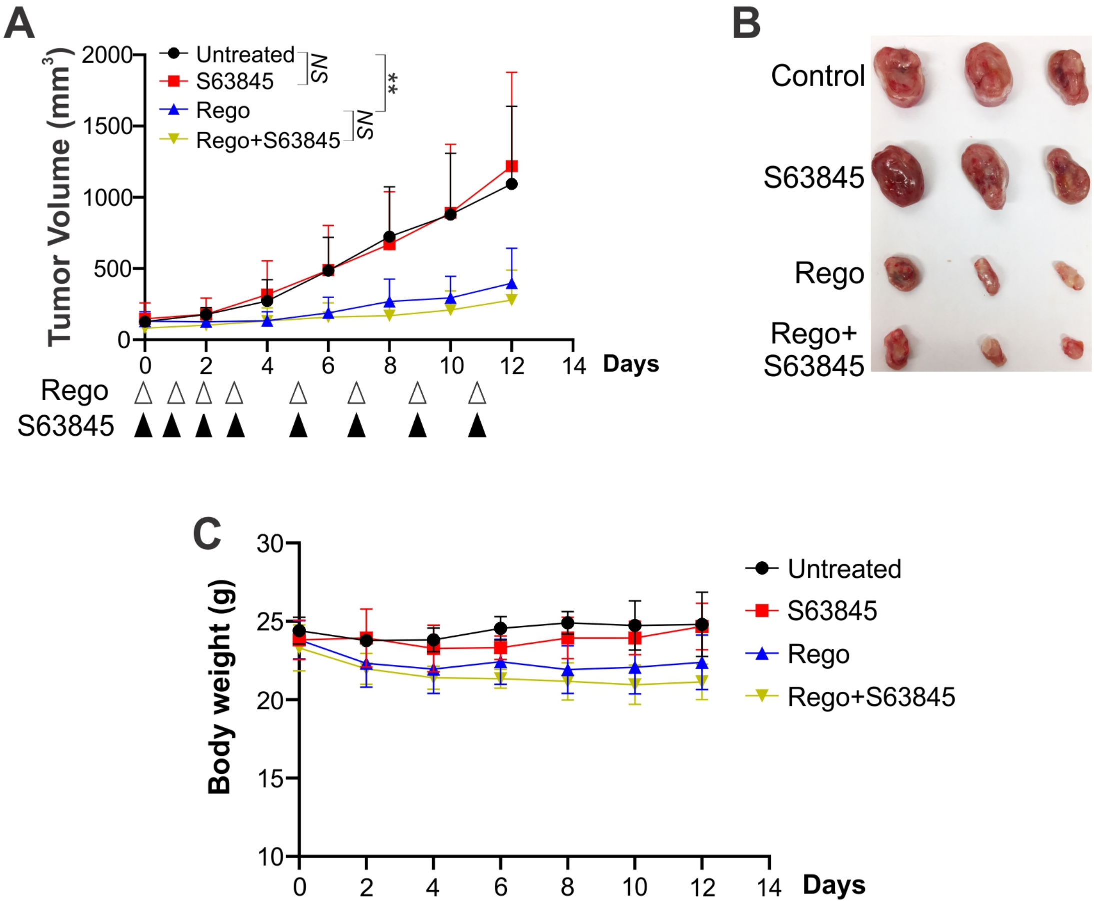

Figure S6. Effects of the regorafenib/S63845 combination in the CT26 syngeneic tumor model. (**A**) BABL/cJ mice were injected subcutaneously with 5×10^5 WT CT26 cells. After tumor growth for 7 days, mice were treated with regorafenib (oral gavage; 20 mg/kg), S63845 (i.p.; 20 mg/kg), or their combination with S63845 (i.p.; 20 mg/kg) as indicated. Tumor volume at indicated time points after treatment was calculated and plotted (n=6-8 in each group). (**B**) Representative pictures of tumors at the end of the experiment in **A**. (**C**) Body weight of tumor-bearing mice at indicated time points. *NS*, *P* > 0.05; ******, *P* <0.01.

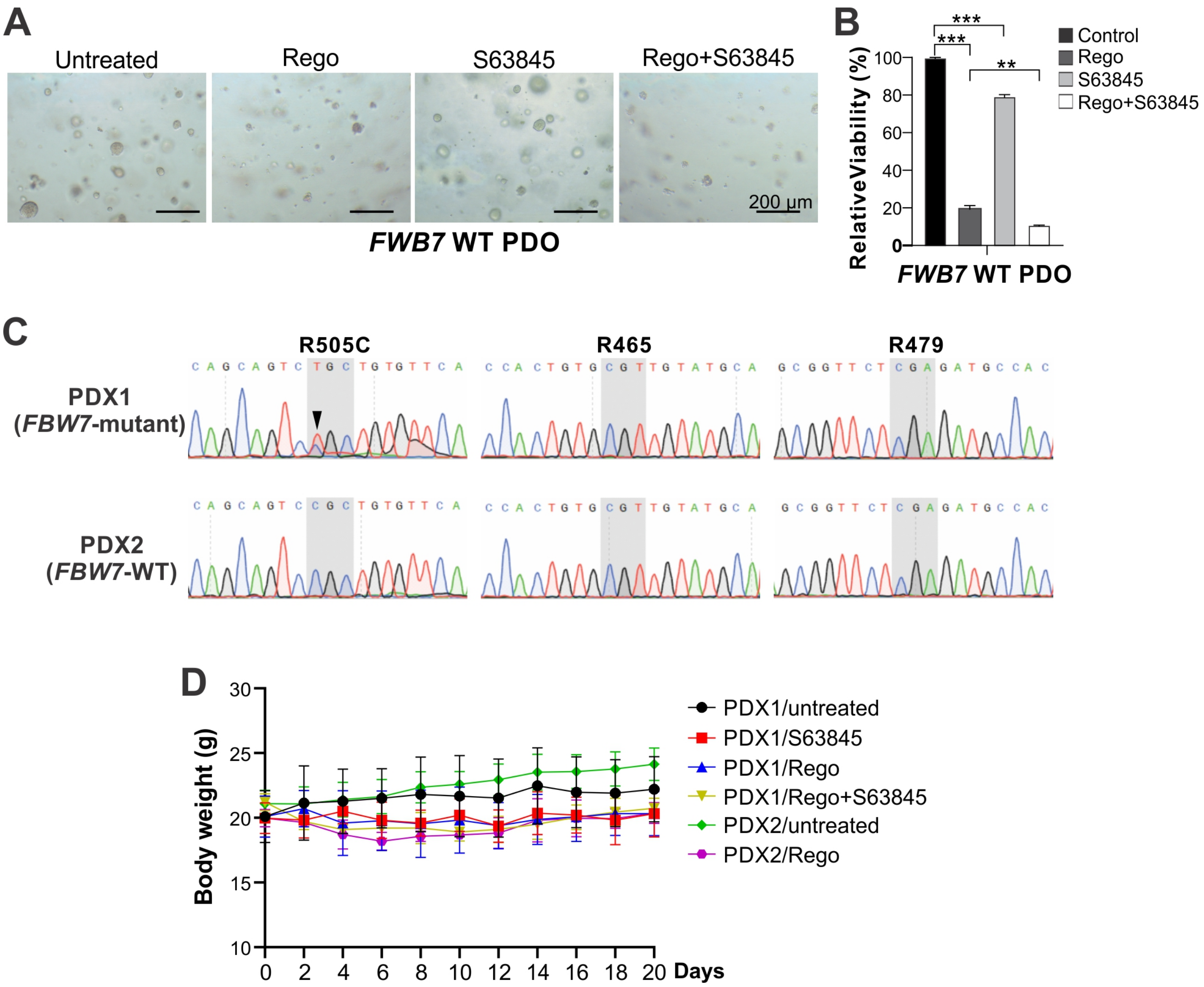

Figure S7. Effects of the regorafenib/S63845 combination in patient-derived models. (**A**) and (**B**) Representative images (**A**) and quantification (**B**) of *FBW7*-WT CRC PDO treated with regorafenib (20 µM) alone or in combination with S63845 (1 µM) for 48 hours. Results were expressed as means ± s.d. of three independent experiments. (**C**) DNA sequencing of the targeted genomic regions in indicated patient-derived xenograft (PDX) tumors highlighting WT and corresponding *FBW7* mutant sequences. (**D**) Body weight of NOD/SCID mice (n=6) harboring indicated PDX tumors at indicated time points after treatment with regorafenib (oral gavage; 20 mg/kg) alone or in combination with S63845 (i.p.; 20 mg/kg).
